# Efficacy and safety of *Saccharomyces boulardii* CNCM I-745 for the treatment of pediatric acute diarrhea in China: a systematic review and meta-analysis

**DOI:** 10.3389/fcimb.2025.1587792

**Published:** 2025-06-04

**Authors:** Lynne V. McFarland, Tong Li

**Affiliations:** ^1^ McFarland Consulting, Seattle, WA, United States; ^2^ Public Health Reserves Corps, Seattle, WA, United States; ^3^ Department of Gastroenterology, First Affiliated Hospital of Dalian Medical University, Dalian, China

**Keywords:** *Saccharomyces boulardii*, pediatric, acute diarrhea, meta-analysis, probiotic

## Abstract

**Background:**

Pediatric acute gastroenteritis (PAGE) is a common cause of morbidity and mortality, especially in children under five years old. Therapeutic strategies including probiotics have been investigated, but trials from non-English speaking countries may not be easily accessible.

**Aim:**

To determine the efficacy of *Saccharomyces boulardii* compared to controls for treating PAGE in children receiving standard rehydration therapy in trials conducted in China.

**Methods:**

Systematic review and meta-analysis using literature search with Google Scholar, PubMed, Embase, Cochrane Library, China National Knowledge Infrastructure and China Biology Medicine disc (from inception to June 30, 2024) of randomized, controlled trials comparing *S. boulardii* CNCM I-745 to controls for the treatment of PAGE in children conducted in China. Independent data extraction by two reviewers. Standard meta-analysis methods were applied and random-effect or fixed-effects models were used depending upon the degree of heterogeneity using standardized mean differences for continuous data and relative risk estimates for dichotomous outcomes. The risk of bias for each study was determined and heterogeneity was measured by I^2^.

**Results:**

Of 851 articles screened, 10 RCTs (1125 participants) met the inclusion criteria, and none were found in non-Chinese databases. *S. boulardii* CNCM I-745 was found to significantly reduce the duration of PAGE (SMD=-1.63 days, 95% CI -2.08, -1.18), improve the total effectiveness rating (RR=1.22, 95% CI 1.16, 1.28) and significantly more participants were cured (RR=1.47, 95% CI 1.30, 1.67). The finding that *S. boulardii* significantly reduced the levels of two pro-inflammatory cytokines (TNF-α and IL8) has not been reported in previous meta-analyses of PAGE.

**Conclusion:**

*S. boulardii* CNCM I-745 is an effective treatment for PAGE and was well tolerated in trials done in China.

**Systematic review registration:**

www.crd.york.ac.uk/PRSPERO, identifier CRD 42024567537.

## Introduction

Pediatric acute gastroenteritis (PAGE) is the third leading cause of morbidity, with more than 1.7 billion cases/year and mortality (nearly 500,000 million deaths/year) worldwide in children under five years old (Aghsaeifard et al., 2022; World Health Organization, 2024). Consequences of PAGE include increased risk of hospitalization and emergency room visits and an increased risk of dehydration and mortality (Zhang et al., 2016; Aghsaeifard et al., 2022). The incidence of PAGE varies globally by country, but also within provinces in China (Wang et al., 2021; World Health Organization, 2024).

Current guidelines for PAGE treatments include oral rehydration therapy (ORT), continued oral feeding and anti-infective medications for viral and bacterial etiologies (Chen et al., 2018). Challenges for these recommendations include the low frequency of ORT use in underdeveloped countries due to poor education for its use, inadequate water quality and limited access to medical resources (Troeger et al., 2020; Aghsaeifard et al., 2022). In addition, to shorten the duration of diarrhea and reduce its severity, several add-on therapies have been recommended, notably racecadotril, diosmectite, and certain probiotics including *Saccharomyces boulardii* CNCM I-745 and *Lactobacillus rhamnosus* GG (Guarino et al., 2018; Szajewska et al., 2023). However, a broad consensus is lacking for which probiotics are more effective due to high study heterogeneity when different probiotic types are pooled in meta-analyses (McFarland et al., 2018; Depoorter and Vandenplas, 2021).

*Saccharomyces boulardii* CNCM I-745 is a widely available probiotic yeast effective for a wide variety of intestinal diseases, including PAGE (McFarland, 2010; Szajewska et al., 2020). The exact mechanisms by which *S boulardii* exerts its actions are multifactorial and include the interference with pathogen attachment, restoration of disrupted intestinal microflora, inactivation of toxins (including *Vibrio cholera*, ETEC, *Clostridioides difficile*, etc.), antisecretory effects and immunomodulatory effects, both within the lumen and systemically (Stier and Bischoff, 2016; Plaza-Diaz et al., 2019). Many relevant clinical trials have been published in China, none of which have been included in published systematic reviews and meta-analyses published in the English literature.

The aim for this meta-analysis is to systematically review evidence on the effects of *S boulardii* CNCM I-745 compared with controls when children are treated with standard rehydration therapies for acute gastroenteritis in trials conducted in China.

## Materials and methods

Our meta-analysis followed the 2020 Preferred Reporting Items for Systematic Reviews and Meta-Analyses guidelines (see **Supplementary Table S1**) and recent recommendations to improve the reporting of probiotic meta-analyses (Page et al., 2021; McFarland et al., 2023). The protocol was prospectively registered with the International Prospective Register of Systematic Reviews (#CRD42024567537) on July 10, 2024 and is available at www.crd.york.ac.uk/PROSPERO/. The protocol was revised October 30, 2024 (more rigorous inclusion and exclusion criteria).

### Search strategy

Open access literature databases [PubMed, Google Scholar, Embase, Cochrane Library, China National Knowledge Infrastructure (CNKI) and China Biology Medicine disc (CBMdisc)] were searched from inception to June 30, 2024, to identify randomized controlled trials (RCT) for the treatment of PAGE with *Saccharomyces boulardii* CNCM I-745 in China. Our search strategies are given in **Supplementary Table S2**. No language restrictions were imposed and publications in Chinese were translated into English. Secondary searches of grey literature included reference lists, authors, reviews, meeting abstracts websites and clinicaltrials.gov for unpublished trials.

### Study screening

Inclusion criteria included: RCT with prospective parallel groups, randomized to either *S. boulardii* CNCM I-745 or control (open or placebo), children (≤18 years old) with acute diarrhea (<15 days) and living in China. Etiologies of acute diarrhea may include viral, bacterial, or unspecified. All participants were allowed conventional therapies (oral or IV rehydration therapy, antiviral medication or antibiotics as needed, diet changes, or correction of acid/base electrolytes). The intervention was a yeast (*Saccharomyces boulardii* CNCM I-745, “Yihuo”, Biocodex, France) given orally. Randomization of the study groups was required to be clearly stated in paper (not just ‘divided into two groups’ or not specified).

Exclusion criteria included: adult patients (>18 years old), studies not done in China, non-human studies, case reports or case series, early phase 1 (safety) or phase 2 (mechanism of action, dose ranging, formulation, kinetics) studies, retrospective case-control studies, no control group, intervention not well-described, no relevant outcomes provided, not a comparison of interest, reviews, meta-analysis, and duplicate reports or did not contain original quantitative data. Trials that gave additional treatments, including specific antibiotics not directed for bacterial diarrhea, zinc, montmorillonite, racecadotril, fructose or aluminum diphosphate, other probiotics, or Chinese medicines were excluded. Other types of diarrhea (AAD, IBS, diarrhea secondary to pneumonia, allergic diarrhea or lientery diarrhea) and trials comparing *S. boulardii* to another type of probiotic without a non-probiotic control group were excluded.

### Study selection and data extraction

Two reviewers (LVM and TL) independently screened titles and abstracts of studies identified by the search strategies. Full articles were translated and screened with inclusion and exclusion criteria. Data from eligible articles were extracted and reviewed independently by two reviewers using a pre-designed data extraction form (see **Supplementary Form S1, DEF**) following guidelines for clinical trials and standard methods for systematic reviews and meta-analysis (Moher et al., 2010; Page et al., 2021; McFarland et al., 2023). Any disagreements were discussed with the reviewers until resolved. The data extracted included PICO data (1): Population (pediatric, age range, country) (2), Intervention (type of *S. boulardii* or controls used, daily doses, formulation, duration and follow-up times) (3), Comparisons (type of control group either placebo or open (4), Outcomes, including improvement in PAGE symptoms (duration of diarrhea, effectiveness rating, cure rate, stool frequency/day by end of study), time to resolution of vomiting or fever, length of hospitalizations, safety measures and changes in immune markers.

To assess sources of heterogeneity and their influence on efficacy, data on potential confounding factors were collected: study design (double blinded or open), risk of bias, study quality, age, setting (inpatient or outpatient) and etiology of diarrhea. For data not reported in the published article, we attempted to contact the author or co-authors to obtain the missing data.

### Study quality

Quality and risk of bias were scored independently by both co-authors using standard methods (Sterne et al., 2001). The risk of bias (RoB) assessed with the RoB 2.0 tool and was graded (low, some concerns or high) for each of five types of bias (randomization process, deviations from intended interventions, missing outcome data, outcome measures, selection of reported result (Sterne et al., 2001). Study quality was also assessed for the 24 recommended items for clinical trials and graded as: high quality (≥75%), moderate quality (12–17 items present, 50-74%), and low quality (<12 items present, <50%). A summary table of risk of bias was generated and the effect of study quality was assessed in trials (McGuinness, 2024). GRADE analysis for selected outcomes was also conducted.

### Primary outcome: resolution of diarrhea

Resolution of diarrhea was measured with four different outcome measures (1): Duration of diarrhea (defined as days of diarrhea from enrollment to the last day of reported diarrheal symptoms) (Aghsaeifard et al., 2022); Effectiveness rating for the improvement of diarrhea symptoms (defined into three categories: ‘markedly effective or cured’, resolution of diarrhea symptoms by the end of the intervention, ‘valid or improved’, moderate improvement in symptoms; and ‘not valid or no effect’, diarrhea symptoms persist at end of intervention, with Total Effectiveness Rating including ‘markedly effective (cured)’ plus ‘valid (improved)’ categories (3); Cured by end of treatment (defined as no diarrhea noted by end of treatment); and (4) Stool frequency per day at end of study treatment. Clinical data was typically recorded by staff if children were hospitalized.

### Secondary outcomes

Four secondary outcomes were analyzed: (1) Frequency of adverse events (safety) during the study; (2) Changes in immune markers (cytokines) from baseline to study end; (3) Reduction in length of stay (if inpatients); (4) Resolution of upper gastrointestinal symptoms (days until vomiting, nausea or abdominal pain cessation). Levels of cytokines were quantified using standard laboratory assays at enrollment and end of intervention.

### Sub-group analyses

Where sufficient data was reported, the efficacy of *S. boulardii* was analyzed by factors that may affect the degree of efficacy including etiology of diarrhea, duration of study intervention, daily dose of *S. boulardii*, risk of bias, study quality, study design (open or placebo controls) and age group.

### Data analysis

Inclusion of studies in meta-analysis required >2 random controlled trials (RCT) using a common outcome measure comparing *S. boulardii* to a non-probiotic control. Statistical analysis and generation of forest plots of pooled summary estimates were performed using Stata software version 16 (Stata Corporation, College Station, Texas) with meta-analysis modules (Palmer and Sterne, 2016). Dichotomous outcomes were assessed using relative risks (RR) and 95% confidence intervals (CI) and continuous outcomes were assessed using standardized mean difference (SMD) and 95% confidence intervals (CI) using standard methods (Higgins et al., 2011). The significance level was set at p-value <0.05. Heterogeneity across trials was evaluated using the I^2^ statistic, 0% indicating none and >50% indicating a high degree of heterogeneity across the trials (Palmer and Sterne, 2016). Bayesian random effects models were used for the meta-analyses when high heterogeneity was found (I^2^>50%), otherwise fixed-effects models were used (Borenstein et al., 2010). Publication bias was assessed using funnel plots and the Egger’s test (Palmer and Sterne, 2016). Subgroup analyses were used to explore sources of heterogeneity and assessed with the Cochrane Q test (Higgins et al., 2011). Sequential sensitivity analysis was done to explore the extent outcomes were dependent upon a particular trial. Trials with missing data for an outcome measure were excluded from the models and no imputation methods were used for missing data.

## Results

### Literature search

The literature search found 851 abstracts using several databases: Google Scholar/PubMed, n=198 and the China National Knowledge Infrastructure (CNKI) and Chinese Biology Medicine disc (CBMdisc), n=653, as shown in **Supplementary Figure S1**. Searches with Embase and the Cochrane Library did not yield any eligible trials. After 193 duplicates were excluded, 658 abstracts were screened and 194 were excluded during initial screening (reviews, meta-analyses, guidelines, meeting abstracts, animal model studies, etc.) and 464 full articles were reviewed. Of these, 274 did not meet inclusion criteria (for example, non-PAGE patients were enrolled, n=123, or *S. boulardii* was used in both control and intervention groups, n=73). Of 190 trials of PAGE, 180 were excluded when additional treatments were used in the trial such as zinc, montmorillonite, racecadotril, etc. (n=109), the control group another type of probiotic (n=39), the strain of *S. boulardii* was not described (n=23), outcome was not diarrhea (n=6) or the study intervention included both *S. boulardii* and another type of probiotic (n=3). Examples of nine trials excluded for commonly found reasons are provided in **Supplementary Table S3** (Li et al., 2014; Duan et al., 2017; Vineeth et al., 2017; Wang et al., 2017; Zhao et al., 2017; Feng et al., 2018; Liu, 2020; Mourey et al., 2020; Altcheh et al., 2022). A total of 10 trials were included in our review (1125 participants) (Qu, 2012; Chen, 2014; Lv, 2014; Tan, 2015; Yang, 2015; Cao et al., 2017; Qiu, 2018; Yao, 2018; Chen, 2020; Wu, 2021). All Chinese articles were translated into English. Publication bias was assessed for the ten RCTs and found publication bias was present (p=0.016), due to more trials with a higher effectiveness rating, as shown in **Supplementary Figure S2**.

### Study participant characteristics

The characteristics of the trials and study participants are provided in **Supplementary Table S4**. All trials were conducted in children (<1 month to 6 years old) with acute diarrhea. The children were enrolled shortly after the onset of the diarrhea: <2–5 days (7, 70%), <15 days (1, 10%), but the specific day of diarrhea onset was not reported in two trials (Qu, 2012; Wu, 2021). The most identified etiology of PAGE was rotavirus (4, 40%) (Chen, 2014; Tan, 2015; Yang, 2015; Chen, 2020), one trial enrolled children with bacterial diarrhea (1, 10%) (Qiu, 2018) and 5 (50%) did not report PAGE etiology. Most of the children were inpatients (9, 90%) and one trial enrolled only outpatients (Chen, 2014).

### Study design

The study size ranged widely from 84–188 participants/trial (mean 112.5 ± 33.4), as shown in **Supplementary Table S4**. All ten trials provided conventional therapies as needed to both *S. boulardii* and control groups. All used open controls and none used a placebo. None of the trials reported lost-to-follow-up or attrition in their trials.

Only one trial had a high risk of bias (Tan, 2015), nine were rated as having some concerns and none had a low risk of bias. The most common reasons for low scores were missing items relating to the method of randomization (5, 50% did not provide this data), 100% were not blinded (allocator, outcome assessor and study staff), failure to provide a sample size calculation and failure to discuss limitations, generalization, and comparison with other studies in the discussion. The trial rated with a high risk of bias also did not report a comparison of baseline characteristics in their trial (Tan, 2015). The risk of bias was similar for the domains in most trials, as shown in **Supplementary Table S5**.

### Intervention characteristics

All trials identified the strain of probiotic used as *S. boulardii* CNCM I-745 (Biocodex or ‘Yihuo’) in a sachet. The duration of treatment ranged from 3 days (n=4, 40%), 4–5 days (n=2, 20%) to 7 days (n=3, 30%) but was not reported in one trial (Wu, 2021). None of the trials followed patients after the study intervention was discontinued. The dose of *S. boulardii* ranged from 125 mg/d to 500 mg/d and 7 (70%) trials used different daily doses depending upon the age of the child, while two trials gave a consistent dose regardless of the age (500 mg/d) (Qiu, 2018; Wu, 2021) and one trial did not report the dose of *S. boulardii* given (Cao et al., 2017).

### Primary outcome: reduction in duration of diarrhea

**Figure 1:**
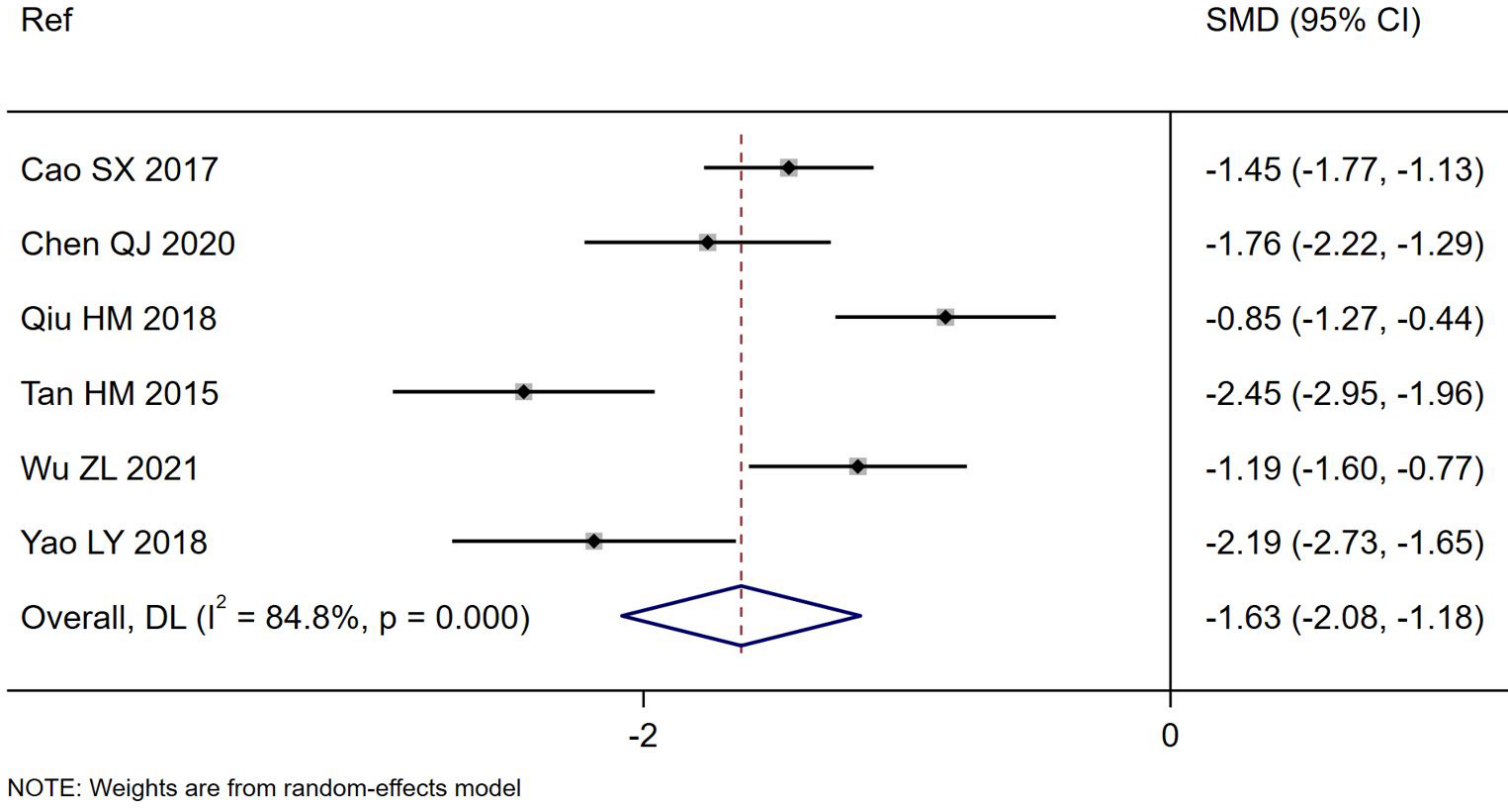
Forest plot of the reduction in duration of PAGE diarrhea (days) comparing *S. boulardii* CNCM I-745 and control groups. CI, confidence interval; DL, DerSimonian-Laird estimate; Ref, reference.

Overall, significant reduction in the duration of days of diarrhea was noted for *S. boulardii* CNCM I-745 compared to the controls (**Table 1**) in six RCT (Tan, 2015; Cao et al., 2017; Qiu, 2018; Yao, 2018; Chen, 2020; Wu, 2021) with the *S. boulardii* group having a mean duration of diarrhea 1.6 days less than the controls (SMD = -1.63 days, 95% CI -2.08, -1.18, z=- 7.05, p<0.0001, I^2^ = 84.8%), as shown in **Figure 1**. Cao et al. reported a very short duration of diarrhea, which could not be confirmed as no contact information was provided (Cao et al., 2017). When this trial was excluded from the analysis, the reduction in mean duration of diarrhea was not significantly different than when this trial was included (SMD: -1.67, 95% CI -2.26, -1.08). When a trial rated with high risk of bias was excluded from the analysis (Tan, 2015), the reduction of days of diarrhea by *S. boulardii* was similar in those trials with moderate rating of risk of bias (SMD= -1.46 days, 95% CI -1.87, -1.06, z=- 7.13, p< 0.001, I^2^ = 77.9%). Other sensitivity analyses found no one trial had an undue influence on the pooled efficacy estimates.

**Table 1:** Pediatric acute gastroenteritis (PAGE) outcome measures comparing *S. boulardii* CNCM I-745 and control groups.

Reference	Duration diarrhea days in Sb (x ± SD)	Duration diarrhea day in controls (x ± SD)	Total effective rate, Sb (%)	Total effective rate, control (%)	Cured in Sb (%)	Cured in controls (%)
Cao SX 2017 (Cao et al., 2017)	0.13 ± 0.05	0.21 ± 0.06	89/94 (94.7)	77/94 (81.9)	63/94 (67.0)	32/94 (34)
Chen LL 2014 (Chen, 2014)	Nr	Nr	40/42 (95.2)	33/42 (78.6)	24/42 (57.1)	20/42 (47.6)
Chen QJ 2020 (Chen, 2020)	2.21 ± 0.69	3.94 ± 1.21	47/49 (95.9)	38/49 (77.5)	24/49 (49.0)	12/49 (24.5)
Lv CG 2014 (Lv, 2014)	Nr	Nr	39/43 (90.7)	30/42 (71.4)	26/43 (60.0)	18/42 (42%)
Qiu HM 2018 (Qiu, 2018)	1.82 ± 1.5	3.05 ± 1.38	45/48 (93.7)	35/48 (73.0)	19/48 (39.6)	14/48 (29.2)
Qu YH 2012 (Qu, 2012)	Nr	Nr	50/55 (90.9)	40/55 (72.7)	29/55 (52.7)	19/55 (34.5)
Tan HM 2015 (Tan, 2015)	2.8 ± 0.63	4.5 ± 0.75	54/55 (98.2)	46/55 (83.6)	48/55 (87.2)	35/55 (63.0)
Wu ZL 2021 (Wu, 2021)	2.75 ± 1.19	4.92 ± 2.33	50/51 (98.0)	43/51 (84.3)	23/51 (45.1)	20/51 (39.2)
Yang XH 2015 (Yang, 2015)	Nr	Nr	46/48 (95.8)	38/48 (79.2)	28/48 (95.8)	18/48 (37.5)
Yao LY 2018 (Yao, 2018)	2.11 ± 0.19	4.8 ± 0.31	61/63 (96.8)	22/30 (73.3)	42/63 (66.7)	13/30 (43.3)

Nr, not reported in paper; Sb, *Saccharomyces boulardii* CNCM I-745; x ± SD, mean ± standard deviation.

Subgroup analysis by etiology of PAGE (rotaviral vs bacterial or not defined) showed a significant difference by etiology (Chi^2^ = 10.6, p=0.005), with a significantly shorter duration of diarrhea noted for those with rotaviral diarrhea (SMD= -2.10 days, 95% CI -2.78, -1.42, p=0.04, I^2^ = 75.1%). The effectiveness in bacterial etiology could not be determined as only one trial included this etiology (Qiu, 2018). A sub-group analysis by the daily dose of *S. boulardii* resulted in lower heterogeneity (**Supplementary Figure S3**). Most trials adjusted the dose (125–500 mg/d) depending upon the age of the child and two trials used a consistent dose (500 mg/d) regardless of the children’s ages. When the dose was age-adjusted, the duration of diarrhea was significantly reduced (SMD= -2.11 days, 95% CI -2.40, -1.83, I^2^ = 51.5%). When the dose was 500 mg/d, the duration of diarrhea was also shortened, but to a lesser extent (SMD= -1.02 days, 95% CI -1.32, -0.73, I^2^ = 18.7%). A sub-group analysis by the duration *S. boulardii* was given resulted in lower heterogeneity, but conclusions could not be drawn due to the limited number of trials with the same number of days of *S. boulardii* given (**Supplementary Figure S4**).

### Primary outcome: total effectiveness rating

**Figure 2:**
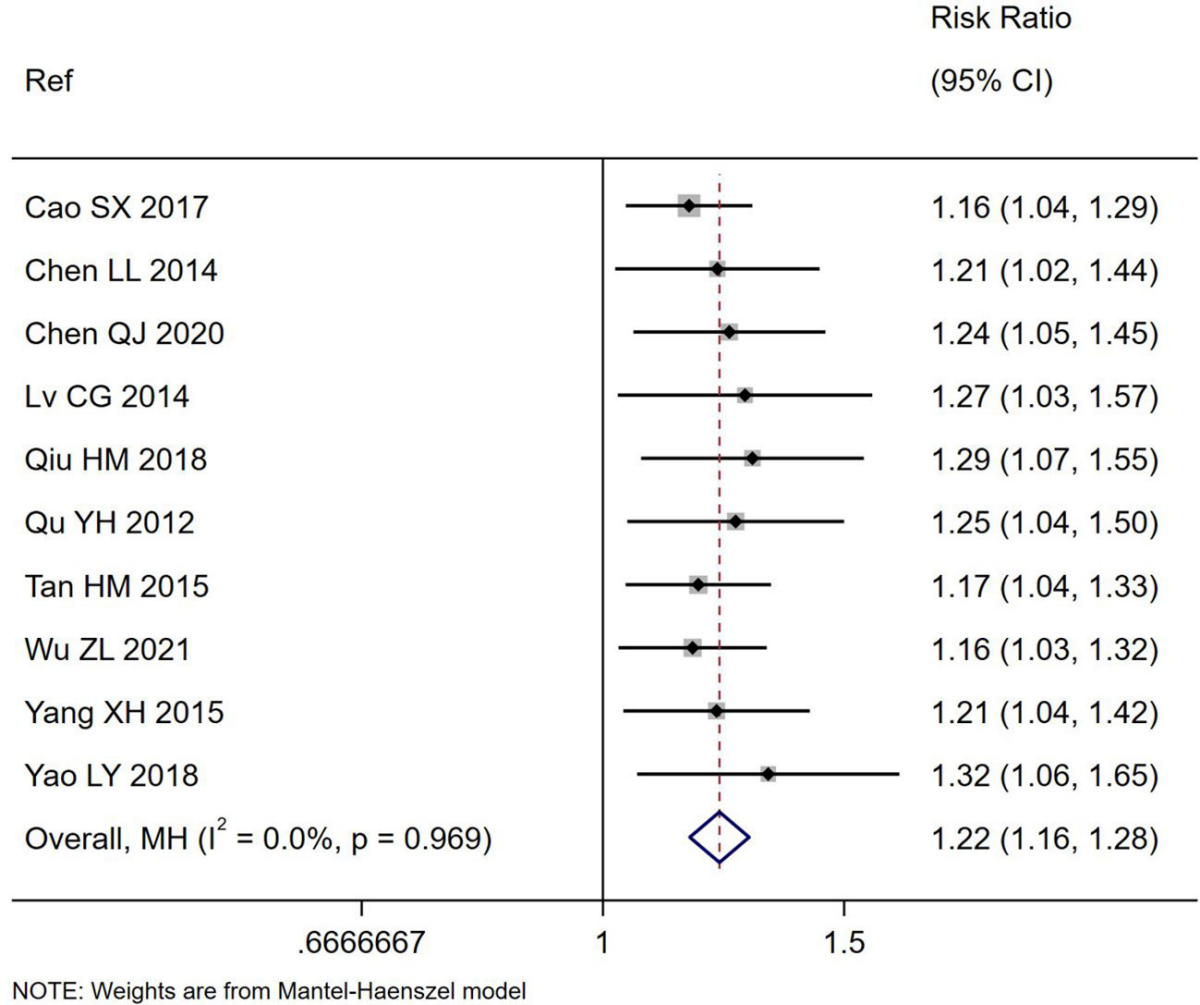
Forest plot of the total effectiveness rating for the improvement of diarrhea symptoms comparing *S. boulardii* CNCM I-745 and control groups. CI, confidence interval; MH, Mantel-Haenszel; Ref, reference.

All ten trials reported the total effectiveness rating for their trials (**Table 1**), which is based on the improvement of diarrheal symptoms. The total effectiveness rating was significantly higher for those treated with *S. boulardii* compared to controls (RR=1.22, 95% CI 1.16, 1.28, z=7.7, p<0.001, I^2^ = 0%), as shown in **Figure 2**. When sub-group analysis was done for different etiologies of PAGE or duration of *S. boulardii* given, neither variable had a significant impact on the improvement of diarrhea symptoms. Different durations of *S. boulardii* given (from 3–7 days) did not significantly impact the effectiveness (Chi^2^ = 1.14, p=0.77). When the one trial with high risk of bias was excluded (Tan, 2015), the nine trials with ‘some concerns’ of risk also reported a higher effectiveness rating in those with *S. boulardii* compared to controls (RR= 1.22, 95% CI 1.16, 1.29, Z=7.28, p<0.001, I^2^ = 0%).

**Figure 3:**
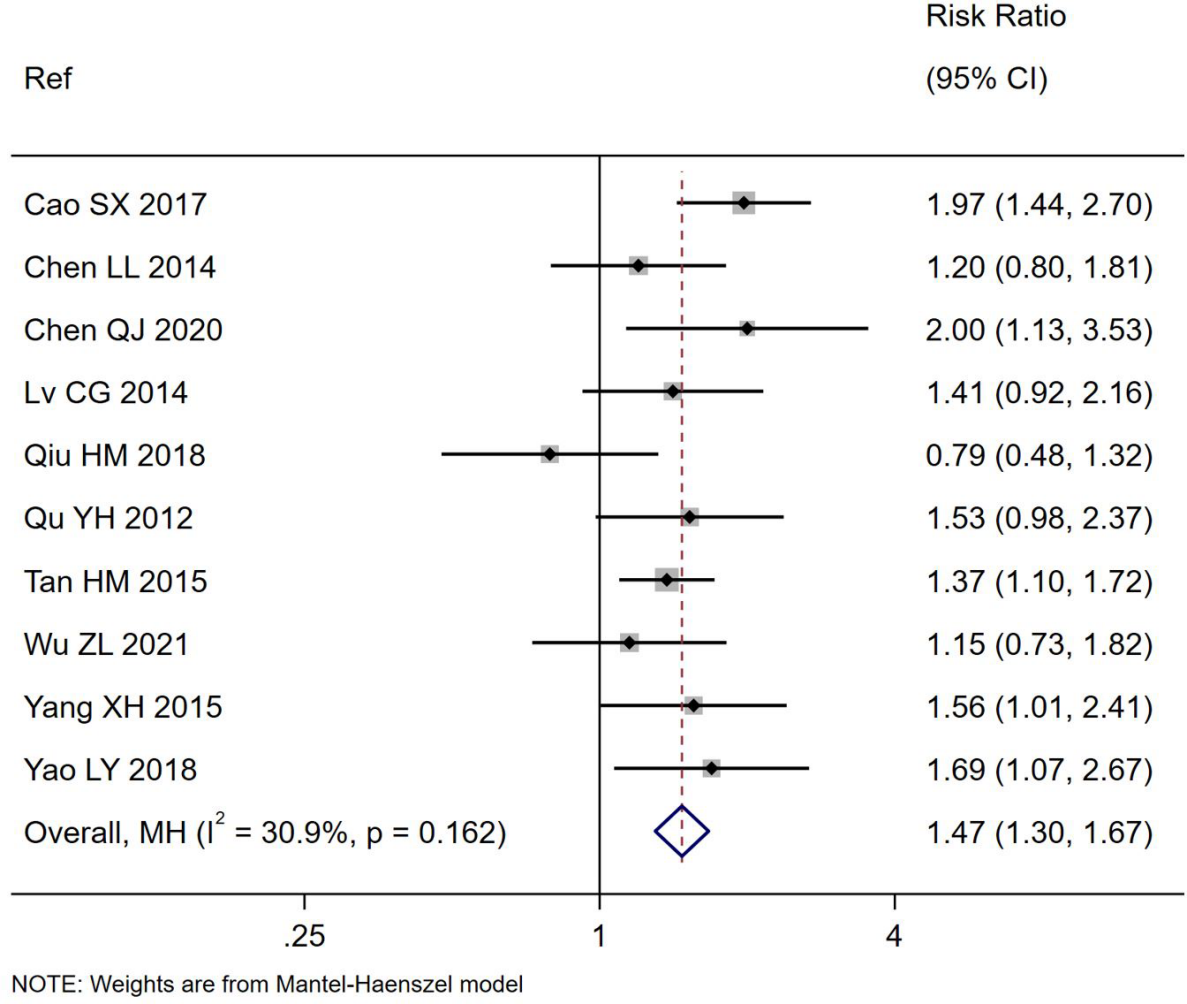
Forest plot of the number cured of PAGE by study end comparing *S. boulardii* CNCM I-745 and control groups. CI, confidence interval; MH, Mantel-Haenszel; Ref, reference.

All ten trials also reported the frequency of cure by the end of the study for their trials (**Table 1**) and significantly more of those treated with *S. boulardii* reported being cured compared to controls (RR= 1.47, 95% CI 1.30,1.67, z=6.04, p<0.0001, I^2^ = 30.9%). as shown in **Figure 3**. There was a significantly higher cure rate in those with rotaviral PAGE (RR=1.46, 95% CI 1.21, 1.75, p=0.03, I^2^ = 0%). The cure rate was similar regardless of the duration of *S. boulardii* given (from 3–7 days), Chi^2^ = 3.61, p=0.31.

### Primary outcome: reduction in stool frequency by end of treatment

A pooled analysis was not possible, as only one trial reported daily stool frequency at the end of treatment (Qiu, 2018). In this trial, those with treated with *S. boulardii* had significantly fewer mean stools per day on Day 3 (1.82 ± 0.54/day, p<0.05) compared to controls (2.93 ± 0.4/d) (Qiu, 2018).

### Secondary outcome: safety

Eight (80%) of the trials reported safety data, while 2 (20%) failed to provide any safety data (Yang, 2015; Qiu, 2018), as shown in **Supplementary Table 4**. Most trials (50%) just provided a statement that ‘no adverse events were reported’, while three trials provided more details for the frequency of reported adverse events by study group (Yao, 2018; Chen, 2020; Wu, 2021). The risk of adverse events was significantly less in those given *S. boulardii* (RR=0.19, 95% CI 0.07, 0.50, p=0.001, I^2^ = 0%) compared to the controls, as shown in **Supplementary Figure S5**. Most of the adverse events were mild, self-resolving symptoms (rash, abdominal discomfort, dry mouth, nausea, vomiting, fever, constipation) and no serious adverse events were reported.

### Secondary outcome: changes in immune markers

**Figure 4:**
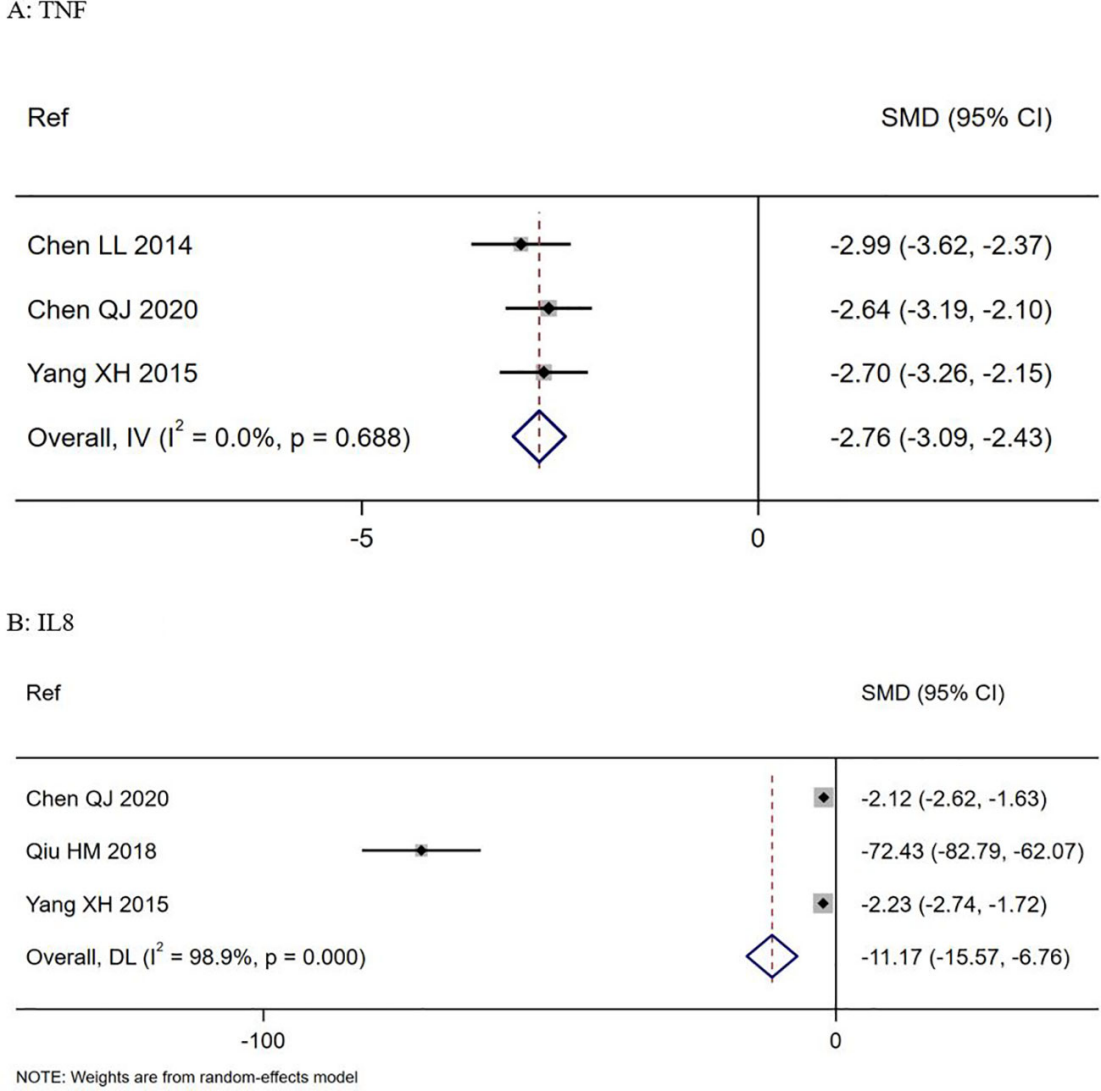
Forest plots of: Top **(A)** Changes in TNF-alpha levels in *S. boulardii* CNCM I-745 compared to controls, Bottom **(B)** changes in IL-8 levels in *S. boulardii* CNCM I-745 compared to controls. CI, confidence interval; DL, DerSimonian-Laird estimate; IV, inverse variance estimate; Ref, reference; SMD, standardized mean difference.

Three trials documented change in TNF-α levels from baseline to study end (Chen, 2014; Yang, 2015; Chen, 2020). Those treated with *S. boulardii* showed a significant reduction in mean TNF-α values compared to controls (SMD = -2.76, 95% CI -3.09, -2.43 z=-16.4, p<0.0001, I^2^ = 0%), as shown in **Figure 4A**. Three trials documented a significant reduction in IL-8 levels from baseline to study end (SMD= -11.2, 95% CI -15.6, -6.8, z=-12.43, p<0.001, I^2^ = 98.9%), as shown in **Figure 4B** (Yang, 2015; Qiu, 2018; Chen, 2020). Although four other trials (Chen, 2014; Cao et al., 2017; Qiu, 2018; Chen, 2020) reported significant changes in other immune markers (CD4/CD8 ratios, CRP, CD3, IL10) for *S. boulardii* compared to controls, no two trials reported the same immune markers so these could not be analyzed (see **Supplementary Table S6**).

### Other secondary outcomes

Several secondary outcomes could not be assessed due to the limited number of trials reporting those outcomes. Only two trials reported time to cessation of vomiting (Chen, 2020; Wu, 2021), only one trial reported time to cessation of nausea (Chen, 2020) and only trial reported the length of hospitalization (Yao, 2018).

## Discussion

Our review found *S. boulardii* CNCM I-745 significantly reduced the duration of PAGE, significantly improved diarrheal symptoms and had a higher cure rate compared to controls in patients in China. This is the first meta-analysis that identified multiple trials in China for this condition using a defined probiotic (*S. boulardii* CNCM I-745). Previous meta-analyses of *S. boulardii* for PAGE treatment did not include any trials from China (Feizizadeh et al., 2014; Szajewska et al., 2020; Huang et al., 2021; Juangco et al., 2021; Li et al., 2021; Fu et al., 2022), which may be due to reliance on standard literature databases, which do not typically include trials published in Chinese language journals. All our included trials were identified in Chinese literature databases and were not detected in Google Scholar or PubMed literature searches.

One challenge in determining the efficacy of probiotics for PAGE treatment in our meta-analysis was that there were multiple types of outcome measures used in our meta-analysis: 100% reported effectiveness rating, 60% reported duration of diarrhea, while only one reported changes in daily stool frequency (Qiu, 2018). The trials done in China reported an outcome measure not typically reported in trials in other countries: Total Effectiveness Rating, which measured the improvement of diarrheal symptoms and not just the duration of days of diarrhea. Meta-analyses done in trials from other countries have also used different types of outcomes (duration of diarrhea, daily stool frequency, vomiting, etc.) and while duration of diarrhea is the most commonly reported outcome, it was not found in all our trials (Szajewska et al., 2020; Huang et al., 2021; Juangco et al., 2021; Li et al., 2021; Fu et al., 2022). A large meta-analysis of 84 RCT of probiotics for PAGE found 14% did not report this outcome (Li et al., 2021) and similarly another meta-analysis of 29 RCT using *S. boulardii* for PAGE reported 29% did not report duration of diarrhea as an outcome (Szajewska et al., 2020), rather other outcomes were reported. As there is no current consensus on which outcome measure should be the gold standard for PAGE, different outcomes will continue to be challenging when a meta-analysis is performed. Our meta-analysis did find a slightly greater reduction in the mean duration of PAGE diarrhea (-1.63 days) in the trials done in China compared to trials done in other countries (ranging from -0.7 to -1.25 days reduction). One possible explanation for the greater reduction in effect in our study may be that other meta-analyses have included trials that included controls with other anti-diarrheal therapies including zinc or other probiotics (Feizizadeh et al., 2014; Szajewska et al., 2020; Fu et al., 2022), while our controls did not include these adjunctive therapies. Our meta-analysis excluded trials that used other additional treatments (such as montmorillonite or traditional Chinese medicines not typically used in non-Chinese treatments of PAGE) in an effort to have comparable therapies so efficacy could be compared when *S. boulardii* is used in Chinese versus other countries. When *S. boulardii* is added to conventional rehydration therapy, a similar degree of efficacy is seen for the reduction of diarrhea when compared to trials done in other countries.

There are several mechanisms-of-action for *S. boulardii* CNCM I-745 which may help to explain the efficacy for the treatment of PAGE including immune regulation, competition with pathogen attachment sites, production of anti-toxin proteases and anti-secretory effects (Buccigrossi et al., 2014; Stier and Bischoff, 2016; Czerucka and Rampal, 2019). Our meta-analysis documented that *S. boulardii* CNCM I-745 was especially effective for reducing the duration of diarrhea caused by rotavirus and this may be due to the ability of this strain to reduce the secretion of chloride (Buccigrossi et al., 2014; Czerucka and Rampal, 2019). Our meta-analysis is the first to our knowledge reporting on the impact of *S. boulardii* CNCM I-745 on immune cytokines in children with PAGE. One mechanism for the efficacy of *S. boulardii* involves the regulation of immune cytokines, but not all trials collected information on immune markers and of those that did, the types of immune markers assessed were not consistent with each other. Thus, only two cytokines (TNF-α and IL8) were able to be assessed. Previous meta-analyses focused on diarrheal outcomes of PAGE, but did not explore this mechanism (Feizizadeh et al., 2014; Szajewska et al., 2020; Huang et al., 2021; Juangco et al., 2021; Li et al., 2021; Fu et al., 2022).

Of the ten trials done in China in children with PAGE, none reported significantly more adverse events in those given *S. boulardii* CNCM I-745 and no serious adverse events were reported. Previous reviews have also reported that *S. boulardii* CNCM I-745 is well-tolerated in children but also noted that not all trials reported safety data (Feizizadeh et al., 2014; Szajewska et al., 2020).

Our meta-analysis has several strengths, including a robust literature review and use of standardized recommendations for reporting probiotic meta-analyses (McFarland et al., 2023). Use of Chinese databases for clinical trials uncovered a wealth of articles not detected in non-Chinese databases (Google Scholar and PubMed, etc.) and the ability of one of the co-authors to accurately translate these studies from the original Chinese language was invaluable. Another strength was our ability to account for strain-specificity important when analyzing the efficacy of probiotics by limiting our inclusion to one defined strain of *S. boulardii* CNCM I-745. Not all probiotic strains are alike and strain-specific effects for various diseases have been clearly demonstrated (McFarland et al., 2018). Strain-specificity has also been observed with other types of probiotics used for the treatment of PAGE (McFarland et al., 2021; Zhao et al., 2021; Chen et al., 2023; Chen et al., 2024). Another strength is that most of the trials used the same definitions for outcome measures and inclusion criteria using standardized definitions.

Our meta-analysis has several limitations. We found the reported clinical trials in China were of moderate quality, mainly due to the lack information about the method of randomization, the lack of blinding of study personnel and study participants, lack of placebo use, and limited information provided on recruitment procedures. The lack of placebo use in the trials done in China may have introduced a level of bias, but open controls using conventional therapies for PAGE have also been used in other countries. In the meta-analysis of Li et al., 42% of the 84 RCTs did not use placebos and in the meta-analysis by Szajewska et al., 62% of 29 RCTs did not use placebos (Szajewska et al., 2020; Li et al., 2021). The lack of blinding or use of placebos should be considered and evaluated but may not have had a large impact on these trials. One meta-analysis of 29 trials assessed the impact of placebo versus open controls and found a slightly larger reduction of the mean duration of diarrhea in 7 trials when *S. boulardii* was compared to placebo controls (MD: -1.24 days, 95% CI -1.74, -0.70) than when *S. boulardii* was compared to open controls in 16 RCTs (MD: -0.98 days, 95% CI -1.29, -0.68) (Szajewska et al., 2020). Another limitation was the inability to identify all the sources of heterogeneity due to the limited number of trials that reported data that might have been used in sub-group analyses. We were able to reduce the degree of heterogeneity for duration of diarrhea when controlling for several factors, including the dose of *S. boulardii* used and for also showing *S. boulardii* CNCM I-745 was more effective in children with rotaviral PAGE. When the overall quality of the trials are examined, several outcomes have a low GRADE recommendation (see **Supplementary Table S7**). Duration of diarrhea has high heterogeneity (85%) as only six trials reported this outcome, but the degree of variance was reduced when only rotaviral diarrhea was analyzed. The two immune markers (TNF-α and IL-8) have a low GRADE rating, as only three trials reported each of these outcomes. Two outcomes (Total Effectiveness Rating and Cure rate) were rated as moderate GRADE, due to low heterogeneity found for all 10 trials. However, as none of the trials were blinded, potential bias may exist. Another limitation is that only 10 trials met our inclusion criteria, which is also observed when other meta-analyses have restricted their meta-analysis to one type of probiotic or one country to reduce heterogeneity (McFarland et al., 2021; Fu et al., 2022).

Future meta-analyses of probiotics could be improved by following recommendations from recognized experts in the field, including a clear definition of the strain(s) of probiotics assessed and not pooling different types of probiotics together in their analysis (McFarland et al., 2023). The quality of a meta-analysis is dependent upon the quality of the individual trials, so efforts to improve individual trials would be beneficial for future meta-analyses. The low-moderate quality in these trials was typically due to not following CONSORT guidelines for reporting clinical trials including a lack of a complete description of the randomization method or stating how allocation assignments were kept blinded (Moher et al., 2010). Future clinical trials need to report follow-up post-study intervention and use blinded interventions (study staff and study participants), as the use of placebo with standard treatments would reduce possible bias. Use of standardized definitions of primary outcome measures is recommended, as multiple definitions were used (antidiarrheal time, time to diarrhea resolution, etc.). In order to assess if immune regulation is involved in PAGE with *S. boulardii*, future trials should collect consistent data on the same type of immune markers. Although we were able to only include a limited number of trials (n=10) due to strict inclusion/exclusion criteria, the finding there are numerous trials using additional treatments (such as montmorillonite, racecadotril or traditional Chinese medicines) not typically found in non-Chinese trials may be of interest to examine in future studies.

As this analysis was based on trials limited to one country (China), it would be prudent to consider how the generalizability of these efficacy results might vary in countries with different diets, health status and lifestyles or differences due to study design and choice of controls. Implications for clinical practice and healthcare policy of this meta-analysis is *S. boulardii* CNCM I-745 is promising for the treatment of PAGE.

This meta-analysis revealed numerous clinical trials that were undiscovered by prior meta-analyses of *S. boulardii* for the treatment of PAGE by utilizing country-specific literature databases. Our meta-analysis found *S. boulardii* CNCM I-745 is an effective and safe treatment for children with PAGE in China.

## Data Availability

The original contributions presented in the study are included in the article/**Supplementary Material**. Further inquiries can be directed to the corresponding author.

## References

[B1] Aghsaeifard Z. Heidari G. Alizadeh R. (2022). Understanding the use of oral rehydration therapy: A narrative review from clinical practice to main recommendations. Health Sci. Rep. 5, e827. doi: 10.1002/hsr2.827 36110343 PMC9464461

[B2] Altcheh J. Carosella M. V. Ceballos A. D/Andrea L. Jofre S. M. Marotta C. . (2022). Randomized, direct comparison study of *Saccharomyces boulardii* CNCM I-745 versus multi-strained *Bacillus clausii* probiotics for the treatment of pediatric acute gastroenteritis. Med. (Baltimore). 101, e30500. doi: 10.1097/MD.0000000000030500 PMC9646502 36086703

[B3] Borenstein M. Hedges L. V. Higgins J. P. Rothstein H. R. (2010). A basic introduction to fixed-effect and random-effects models for meta-analysis. Res. Syn Methods 1, 97–111. doi: 10.1002/jrsm.12 26061376

[B4] Buccigrossi V. Laudiero G. Russo C. Miele E. Sofia M. Monini M. . (2014). Chloride secretion induced by rotavirus is oxidative stress-dependent and inhibited by *Saccharomyces boulardii* in human enterocytes. PloS One 9, e99830. doi: 10.1371/journal.pone.0099830 24918938 PMC4053528

[B5] Cao S.X. Wang Y. Shu G (2017). The effect of *Saccharomyces boulardii* on cellular immune function in the treatment of infantile diarrhea. Contemp Med. 23, 96–98. doi: 10.3969/j.issn.1009-4393.2017.17.049

[B6] Chen L. L. (2014). Influence and curative effect of *Saccharomyces boulardii* powder on serum interleukin-6 and tumor necrosis factor-α levels of children with rotavirus enteritis. Chin. J. Microecol. 26, 181–187. doi: 10.13381/j.cnki.cjw.201402014

[B7] Chen Q. J. (2020). Effects of Saccharomyces boulardii on serum IL-6, IL-8 and TNF-α levels in children with rotavirus enteritis. Mod Med. Health Res. Electronic J. 4, 31–33.

[B8] Chen K. Jin S. Ma Y. Cai L. Xu P. Nie Y. . (2023). Adjunctive efficacy of *Lactis* XLTG11 for Acute diarrhea in children: A randomized, blinded, placebo-controlled study. Nutrition. 111, 112052. doi: 10.1016/j.nut.2023.112052 37172455

[B9] Chen K. Jin S. Ma Y. Cai L. Xu P. Nie Y. . (2024). Adjudicative efficacy of *Bifidobacterium animalis* subsp. *lactis* BLa80 in treating acute diarrhea in children: a randomized, double-blinded, placebo-controlled study. Euro J. Clin. Nutr. 78, 501–508. doi: 10.1038/s41430-024-01428-6 PMC11182741 38467857

[B10] Chen J. Wan C. M. Gong S. T. Fang F. Sun M. Qian Y. . (2018). Chinese clinical practice guidelines for acute infectious diarrhea in children. World J. Pediatr. 14, 429–436. doi: 10.1007/s12519-018-0190-2 30269306

[B11] Czerucka D. Rampal P. (2019). Diversity of *Saccharomyces boulardii* CNCM I-745 mechanisms of action against intestinal infections. World J. Gastroenterol. 25, 2188–2203. doi: 10.3748/wjg.v25.i18.2188 31143070 PMC6526157

[B12] Depoorter L. Vandenplas Y. (2021). Probiotics in pediatrics. A review and practical guide. Nutrients. 13, 2176. doi: 10.3390/nu13072176 34202742 PMC8308463

[B13] Duan W. Zhou C. Luo M. Zuo X. (2017). Effects of *Saccharomyces boulardii* powder on disease progression of rotavirus gastroenteritis in children. Mod Dig Interv. 22, 692–694. doi: 10.3969/j.issn.1672-2159.2017.05.028

[B14] Feizizadeh S. Salehi-Abargouei A. Akbari V. (2014). Efficacy and safety of *Saccharomyces boulardii* for acute diarrhea. Pediatrics. 134, e176–e191. doi: 10.1542/peds.2013-3950 24958586

[B15] Feng N. Lei Z. Yang H. Hu L. (2018). Clinical efficacy of *Saccharomyces boulardii* combined with mezlocillin in the treatment of children with infectious diarrhea and effect on serum CRP, PCT and IL-8. Chin. J. Integr. Tradit West Med. Dig. 26, 194–197.

[B16] Fu H. Li J. Xu X. Xia C. Pan Y. (2022). Effectiveness and safety of *Saccharomyces boulardii* for the treatment of acute gastroenteritis in the pediatric population: A systematic review and meta-analysis of randomized controlled trials. Comput. Math Methods Med. 2022, 1–10. doi: 10.1155/2022/6234858 PMC9514928 36176742

[B17] Guarino A. Lo Vecchio A. Dias J. A. Berkley J. A. Boey C. Bruzzese D. . (2018). Universal recommendations for the management of acute diarrhea in nonmalnourished children. J. Pediatr. Gastroenterol. Nutr. 67, 586–593. doi: 10.1097/MPG.000000000000205 29901556 PMC7116696

[B18] Higgins J. P. Altman D. G. Gøtzsche P. C. Jüni P. Moher D. Oxman A. D. . (2011). The Cochrane collaboration’s tool for assessing risk of bias in randomised trials. BMJ. 343, d5928. doi: 10.1136/bmj.d5928 22008217 PMC3196245

[B19] Huang R. Xing H. Y. Liu H. J. Chen Z. F. Tang B. B. (2021). Efficacy of probiotics in the treatment of acute diarrhea in children: a systematic review and meta-analysis of clinical trials. Transl. Pediatr. 10, 3248. doi: 10.21037/tp-21-511 35070839 PMC8753473

[B20] Juangco J. R. Ramilo-Cruz N. Y. Cruz R. O. Hidalgo M. C. Floro-Cruz K. Abdon R. V. . (2021). Effectiveness of *Saccharomyces boulardii* on diarrhea, a systematic review and meta-analysis. Health Sci. J. 10, 16–24.

[B21] Li G. Wu Y. Li J. Ma J. X. Zhang M. Zhuang Y. (2014). Clinical research of using *Saccharomyces boulardii* to prevent secondary diarrhea in hospitalized neonates. Chin J. Microecol. 26, 1–3. doi: 10.13381/j.cnki.cjm.201401019

[B22] Li Z. Zhu G. Li C. Lai H. Liu X. Zhang L. (2021). Which probiotic is the most effective for treating acute diarrhea in children? A bayesian network meta-analysis of randomized controlled trials. Nutrients. 13, 4319. doi: 10.3390/nu13124319 34959871 PMC8706888

[B23] Liu T. (2020). Analysis of the clinical effect of treating children with rotavirus gastroenteritis with *Saccharomyces boulardii* and *Bifidobacterium* quadruple live bacteria. Cardiovasc. Dis. Integr. Tradit Chin. West Med. 8, 57–63. doi: 10.16282/j.cnki.cn11-9336/r.2020.34.040

[B24] Lv C. G. (2014). Clinical efficacy of *Saccharomyces boulardii* sachets in children with acute diarrhea. Med. J. Chin People’s Health 26, 62–63. doi: 10.3969/j.issn.1672-0369.014.18.035

[B25] McFarland L. V. (2010). Systematic review and meta-analysis of *Saccharomyces boulardii* in adult patients. World J. Gastroenterol. 16, 2202–2222. doi: 10.3748/wjg.v16.i18.2202 20458757 PMC2868213

[B26] McFarland L. V. Evans C. T. Goldstein E. J. (2018). Strain-specificity and disease-specificity of probiotic efficacy: a systematic review and meta-analysis. Front. Med. 5. doi: 10.3389/fmed.2018.00124 PMC5949321 29868585

[B27] McFarland L. V. Hecht G. Sanders M. E. Goff D. A. Goldstein E. J. Hill C. . (2023). Recommendations to improve quality of probiotic systematic reviews with meta-analyses. JAMA Network Open 6, e2346872. doi: 10.1001/jamanetworkopen.2023.46872 38064222

[B28] McFarland L. V. Srinivasan R. Setty R. P. Ganapathy S. Bavdekar A. Mitra M. . (2021). Specific probiotics for the treatment of pediatric acute gastroenteritis in India: A systematic review and meta-analysis. JPGN Reports. 2, e079. doi: 10.1097/PG9.0000000000000079 37205949 PMC10191489

[B29] McGuinness L. A. 2024 Robvis: An R package and web application for visualising risk-of-bias assessments. Available online at: https://github.com/mcguinlu/robvis (Accessed May 20, 2024).

[B30] Moher D. Hopewell S. Schulz K. F. Montori V. Gøtzsche P. C. Devereaux P. J. . (2010). CONSORT 2010 explanation and elaboration: updated guidelines for reporting parallel group randomised trials. BMJ. 340, c869. doi: 10.1136/bmj.c869 20332511 PMC2844943

[B31] Mourey F. Sureja V. Kheni D. Shah P. Parikh D. Upadhyay U. . (2020). A multicenter, randomized, double-blind, placebo-controlled trial of Saccharomyces boulardii in infants and children with acute diarrhea. Pediatr. Infect. Dis. J. 39, e347–e351. doi: 10.1097/INF.0000000000002849 32796401 PMC7556239

[B32] Page M. J. McKenzie J. E. Bossuyt P. M. Boutron I. Hoffmann T. C. Mulrow C. D. . (2021). The PRISMA 2020 statement: an updated guideline for reporting systematic reviews. BMJ. 372, n160. doi: 10.1136/bmj.n160 33782057 PMC8005924

[B33] Palmer T. M. Sterne J. A. C. (2016). Meta-analysis in Stata: an updated collection from the Stata Journal. 2nd ed. (College Station, Texas: Stata Press).

[B34] Plaza-Diaz J. Ruiz-Ojeda F. J. Gil-Campos M. Gil A. (2019). Mechanisms of action of probiotics. Adv. Nutr. 10, S49–S66. doi: 10.1093/advances/nmy063 30721959 PMC6363529

[B35] Qiu H. M. (2018). The effects of *Saccharomyces boulardii* on the expression of serum IL-8, IL-10 and C-reactive protein in children with infectious diarrhea. J. Ped Pharm. 24, 29–31. doi: 10.13407/j.chki.jpp.1672-108X.2018.04.010

[B36] Qu Y. H. (2012). Clinical efficacy of *Saccharomyces boulardii* in the treatment of acute diarrhea in children. Contemp Medi Symp. 10, 211.

[B37] Sterne J. A. C. Egger M. Smith G. D. (2001). Systematic reviews in health care: Investigating and dealing with publication and other biases in meta-analysis. BMJ. 323, 101–105. doi: 10.1136/bmj.323.7304.101 11451790 PMC1120714

[B38] Stier H. Bischoff S. (2016). Influence of *Saccharomyces boulardii* CNCM I-745 on the gut-associated immune system. Clin. Exp. Gastroenterol. 9, 269–279. doi: 10.2147/CEG.S111003 27695355 PMC5027949

[B39] Szajewska H. Canani R. B. Domellöf M. Guarino A. Hojsak I. Indrio F. . (2023). Probiotics for the management of pediatric gastrointestinal disorders: position paper of the ESPGHAN special interest group on gut microbiota and modifications. J. Ped Gastroenterol. Nutri. 76, 232–247. doi: 10.1097/MPG.0000000000003633 36219218

[B40] Szajewska H. Kołodziej M. Zalewski B. M. (2020). Systematic review with meta-analysis: Saccharomyces boulardii for treating acute gastroenteritis in children-a 2020 update. Aliment Pharmacol. Ther. 51, 678–688. doi: 10.1111/apt.15659 32056266

[B41] Tan H. M. (2015). Analysis of the curative effect of *Saccharomyces boulardii* sachets treat children rotaviral enteritis. World Latest Med. Inform. 15, 26–29. doi: 10.3969/j.issn.1671-3141-2015.70.018

[B42] Troeger C. E. Khalil I. A. Blacker B. F. Biehl M. H. Albertson S. B. Zimsen S. R. . (2020). Quantifying risks and interventions that have affected the burden of diarrhoea among children younger than 5 years: an analysis of the global burden of disease study 2017. Lancet Infect. Dis. 20, 37–59. doi: 10.1016/S1473-3099(19)30401-3 31678029 PMC7340495

[B43] Vineeth S. Saireddy S. Keerthi T. Mmantada K. (2017). Efficacy of *Bacillus clausii* and *Saccharomyces boulardii* in treatment of acute rotaviral diarrhea in pediatric patients. Indonesian J. Clin. Pharm. 6, 91–98. doi: 10.15416/ijcp.2017.6.2.91

[B44] Wang D. Ye Q. Guo Q. (2017). Clinical observation of *Saccharomyces boulardii* adjuvant with aluminium phosphate in the treatment of pediatric acute diarrhea. China Pharm. 28, 3250–3254. doi: 10.6039/j.issn.1001-0408.2017.23.23

[B45] Wang L. P. Zhou S. X. Wang X. Lu Q. B. Shi L. S. Ren X. . (2021). Etiological, epidemiological, and clinical features of acute diarrhea in China. Nat. Commun. 12, 1–12. doi: 10.1038/s41467-021-22551-z 33927201 PMC8085116

[B46] World Health Organization (2024). Diarrhoeal disease. Available online at: https://www.who.int/news-room/fact-sheets/detail/diarrhoeal-disease (Accessed March 27, 2024).

[B47] Wu Z. L. (2021). Analysis of antidiarrheal time and safety of *Saccharomyces boulardii* in children with acute diarrhea. J. North Pharm. 18, 125–126.

[B48] Yang X. H. (2015). Observation of *Saccharomyces boulardii* on serum IL-8 and TNF-α levels in children with rotavirus enteritis and its curative effect. Chin Ped Integrat Trad Western Med. 7, 334–335. doi: 10.3969/j.issn.1674-3865.2015.04.013

[B49] Yao L. Y. (2018). Comparative observation of clinical efficacy of two live *Bacillus subtilis* powder and *Bacillus subtilis* granules in the treatment of acute diarrhea in children. World Latest Medi Inform (Electronic Version). 18, 21–22. doi: 10.19613/j.cnki.1671-3141.2018.57.009

[B50] Zhang S. X. Zhou Y. M. Xu W. Tian L. G. Chen J. X. Chen S. H. . (2016). Impact of co-infections with enteric pathogens on children suffering from acute diarrhea in southwest China. Infect. Dis. Poverty. 5, 1–13. doi: 10.1186/s40249-016-0157-2 27349521 PMC4922062

[B51] Zhao W. Peng C. Sakandar H. A. Kwok L. Y. Zhang W. (2021). Meta-analysis: Randomized trials of *Lactobacillus plantarum* on immune regulation over the last decades. Front. Immunol. 12. doi: 10.3389/fimmu.2021.643420 PMC8019694 33828554

[B52] Zhao Y. F. Shao X. Xu B. Zhou X. Lu Q. C. (2017). Influence of *Saccharomyces boulardii* on expression of serum IL-6 and TNF-α of children with rotavirus infections. Chin. J. Nosocomiol. 27, 4989–4991. doi: 10.11816/cn.ni.2017-170-946

