# Supplementary material for: Efficacy and safety of Saccharomyces boulardii CNCM I-745 for the treatment of pediatric acute diarrhea in China: a systematic review and meta-analysis
Source: Front Cell Infect Microbiol. 2025 Jun 4;15:1587792. doi: 10.3389/fcimb.2025.1587792 (PMC12174131; doi:10.3389/fcimb.2025.1587792)
Supplement: Supplementary file 1 [file Table1.docx]

**Supplementary Material**

Supplementary Figure S1 PRISMA 2020 Study Flow-chart

Supplementary Figure S2. Publication bias

Supplementary Figure S3. Forest plot of duration of diarrhea by dose sub-groups

Supplementary Figure S4. Forest plot of duration of diarrhea by *S. boulardii* duration sub-groups

Supplementary Figure S5. Forest plot of risk of adverse events

Supplementary Table S1. PRISMA 2020 Checklist

Supplementary Table S2. Literature search strategies and key words

Supplementary Table S3. Excluded trials

Supplementary Table S4. Study population and intervention characteristics

Supplementary Table S5. Risk of bias for each included trial

Supplementary Table S6. Inflammatory marker outcomes

Supplementary Table S7. GRADE recommendations for selected outcomes

Supplementary Form S1. Data Extraction Form


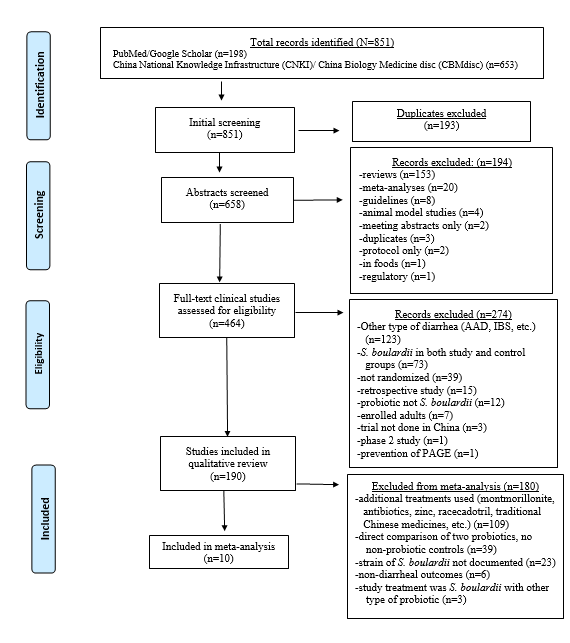


**Supplementary Figure S1**. PRISMA 2020 Flow-chart

**Abbreviations**: AAD, antibiotic-associated diarrhea; Bifido, *Bifidobacterium*; CNKI, China Knowledge Infrastructure; CMBdisc, China Biology Medicine disc; IBS, irritable bowel syndrome; S. *Saccharomyces*.


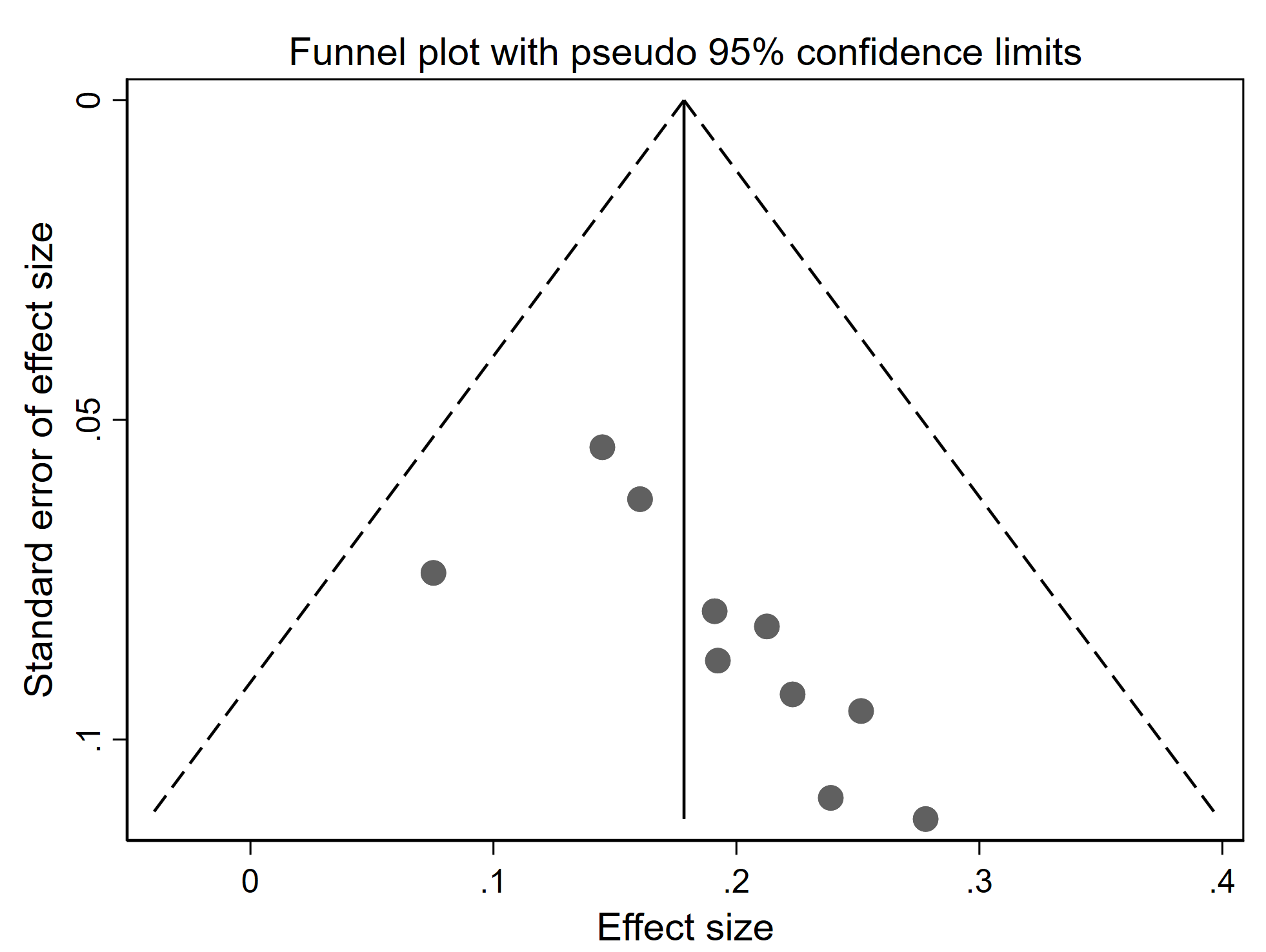


**Supplementary Figure S2.** Publication bias for 10 trials evaluating Total Effectiveness Rating of *S. boulardii* compared to controls.


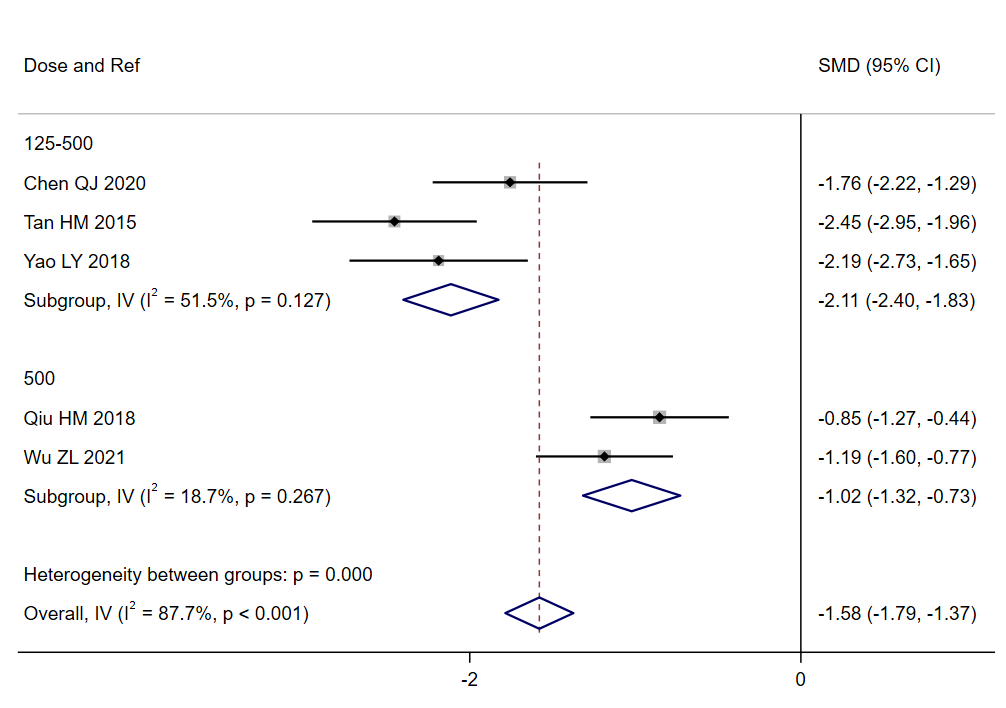


**Supplementary Figure S3**. Forest plot of duration of diarrhea for *S. boulardii c*ompared to controls by dose sub-groups (mg/day). Note 125-500 mg/d group included doses of *S. boulardii* adjusted by different age groups. **Abbreviations**: CI, confidence interval; IV, inverse variance; .Ref, reference; SMD, standardized mean difference.


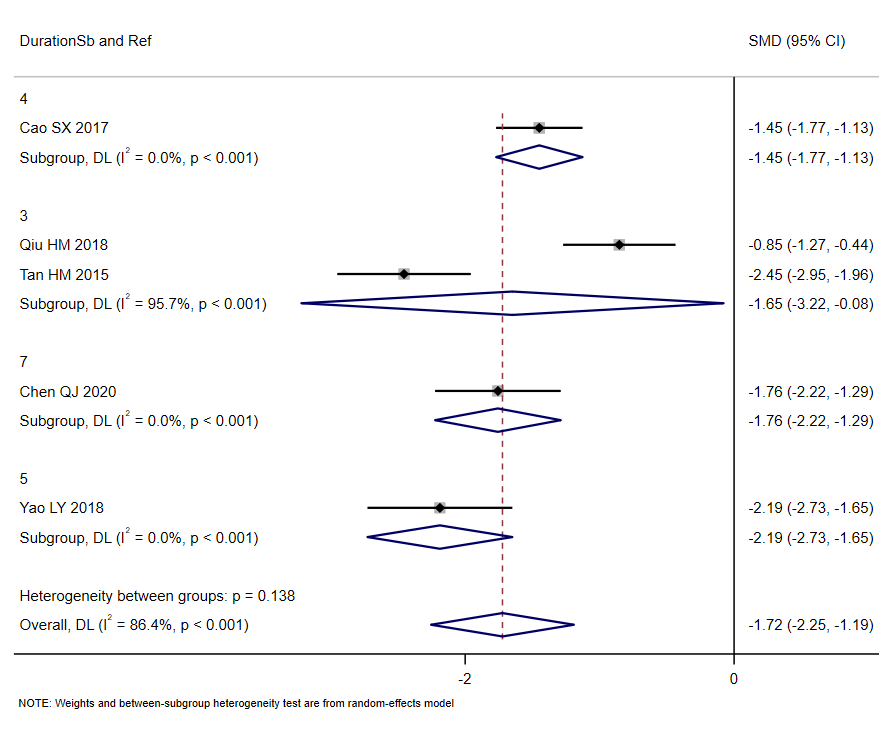


**Supplementary Figure S4**. Forest plot of duration of diarrhea (days) for *S. boulardii* compared to controls by days *S. boulardii* given. **Abbreviations**: CI, confidence interval; DL, DerSimonian-Laird estimate; Ref, reference; SMD, standardized mean difference.


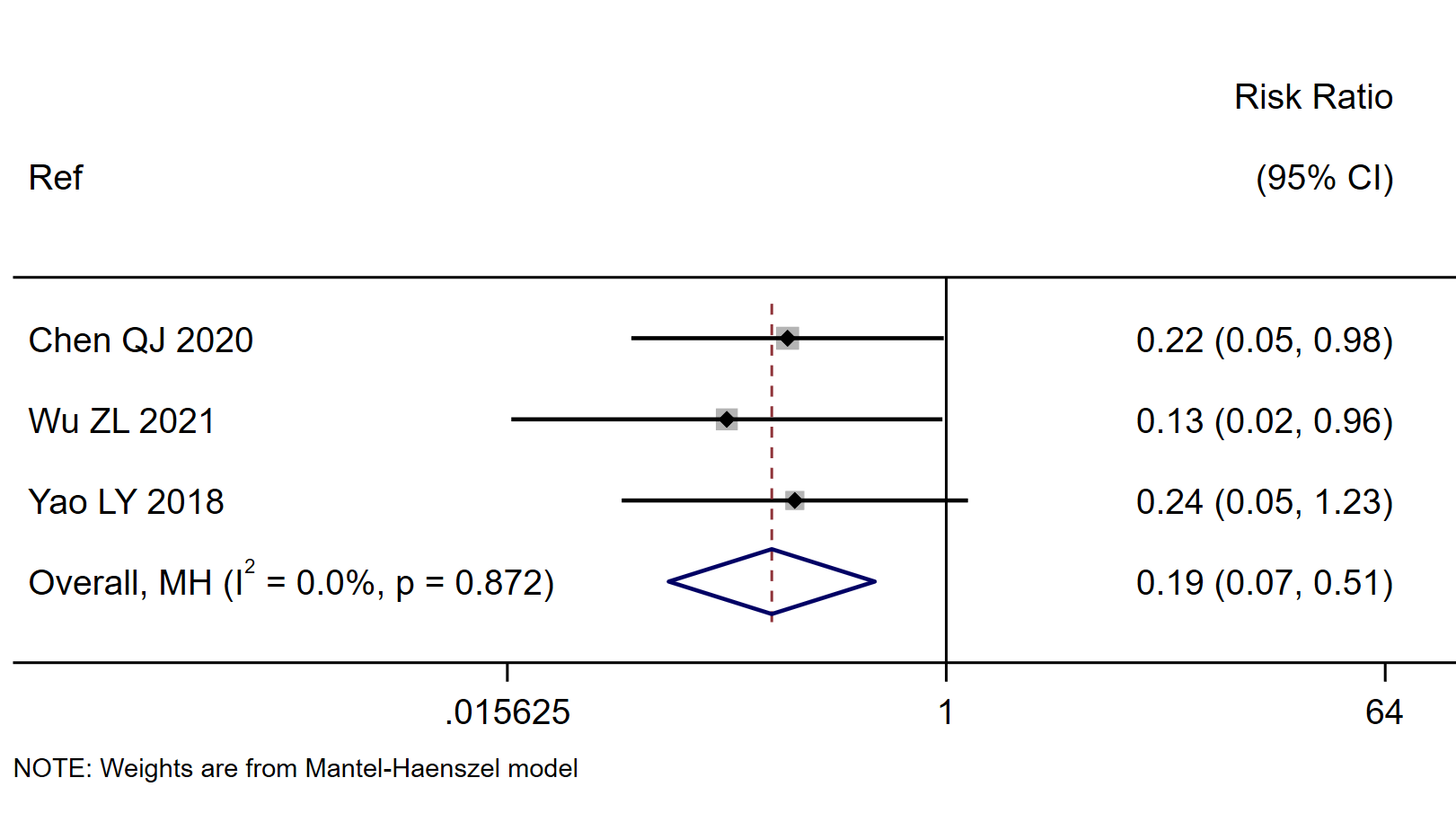


**Supplementary Figure S5**. Forest plot of risk of any reported adverse events for *S. boulardii* CNCM I-745 compared to controls. **Abbreviations**: CI, confidence interval; Ref, reference

**Supplementary Table S1.** PRISMA 2020 Checklist

| **Section and Topic** | **Item #** | **Checklist item** | **Location where item is reported** |  |
| --- | --- | --- | --- | --- |
| **TITLE** | | |  | |
| Title | 1 | Identify the report as a systematic review. | 1 |  |
| **ABSTRACT** | | |  | |
| Abstract | 2 | See the PRISMA 2020 for Abstracts checklist. | 1 |  |
| **INTRODUCTION** | | |  | |
| Rationale | 3 | Describe the rationale for the review in the context of existing knowledge. | 2 |  |
| Objectives | 4 | Provide an explicit statement of the objective(s) or question(s) the review addresses. | 2 |  |
| **METHODS** | | |  | |
| Eligibility criteria | 5 | Specify the inclusion and exclusion criteria for the review and how studies were grouped for the syntheses. | 3 |  |
| Information sources | 6 | Specify all databases, registers, websites, organisations, reference lists and other sources searched or consulted to identify studies. Specify the date when each source was last searched or consulted. | 2-3 |  |
| Search strategy | 7 | Present the full search strategies for all databases, registers and websites, including any filters and limits used. | Supp Table S2 |  |
| Selection process | 8 | Specify the methods used to decide whether a study met the inclusion criteria of the review, including how many reviewers screened each record and each report retrieved, whether they worked independently, and if applicable, details of automation tools used in the process. | 3 |  |
| Data collection process | 9 | Specify the methods used to collect data from reports, including how many reviewers collected data from each report, whether they worked independently, any processes for obtaining or confirming data from study investigators, and if applicable, details of automation tools used in the process. | 3 Supp Form S1 |  |
| Data items | 10a | List and define all outcomes for which data were sought. Specify whether all results that were compatible with each outcome domain in each study were sought (e.g. for all measures, time points, analyses), and if not, the methods used to decide which results to collect. | 4 |  |
|  | 10b | List and define all other variables for which data were sought (e.g. participant and intervention characteristics, funding sources). Describe any assumptions made about any missing or unclear information. | 3 |  |
| Study risk of bias assessment | 11 | Specify the methods used to assess risk of bias in the included studies, including details of the tool(s) used, how many reviewers assessed each study and whether they worked independently, and if applicable, details of automation tools used in the process. | 3-4 |  |
| Effect measures | 12 | Specify for each outcome the effect measure(s) (e.g. risk ratio, mean difference) used in the synthesis or presentation of results. | 4-5 |  |
| Synthesis methods | 13a | Describe the processes used to decide which studies were eligible for each synthesis (e.g. tabulating the study intervention characteristics and comparing against the planned groups for each synthesis (item #5)). | 3 |  |
|  | 13b | Describe any methods required to prepare the data for presentation or synthesis, such as handling of missing summary statistics, or data conversions. | 3 |  |
|  | 13c | Describe any methods used to tabulate or visually display results of individual studies and syntheses. | 4-5 |  |
|  | 13d | Describe any methods used to synthesize results and provide a rationale for the choice(s). If meta-analysis was performed, describe the model(s), method(s) to identify the presence and extent of statistical heterogeneity, and software package(s) used. | 4-5 |  |
|  | 13e | Describe any methods used to explore possible causes of heterogeneity among study results (e.g. subgroup analysis, meta-regression). | 3-4 |  |
|  | 13f | Describe any sensitivity analyses conducted to assess robustness of the synthesized results. | 5 |  |
| Reporting bias assessment | 14 | Describe any methods used to assess risk of bias due to missing results in a synthesis (arising from reporting biases). | 5 |  |
| Certainty assessment | 15 | Describe any methods used to assess certainty (or confidence) in the body of evidence for an outcome. | 4-5 |  |
| **RESULTS** | | |  | |
| Study selection | 16a | Describe the results of the search and selection process, from the number of records identified in the search to the number of studies included in the review, ideally using a flow diagram. | 5, Supp Fig S1 |  |
|  | 16b | Cite studies that might appear to meet the inclusion criteria, but which were excluded, and explain why they were excluded. | 5, Supp Table S3 |  |
| Study characteristics | 17 | Cite each included study and present its characteristics. | Supp Table S4 |  |
| Risk of bias in studies | 18 | Present assessments of risk of bias for each included study. | Supp Table S5 |  |
| Results of individual studies | 19 | For all outcomes, present, for each study: (a) summary statistics for each group (where appropriate) and (b) an effect estimate and its precision (e.g. confidence/credible interval), ideally using structured tables or plots. | Fig 1-4,  Table 1 |  |
| Results of syntheses | 20a | For each synthesis, briefly summarise the characteristics and risk of bias among contributing studies. | 5-6, Supp Table S5 |  |
|  | 20b | Present results of all statistical syntheses conducted. If meta-analysis was done, present for each the summary estimate and its precision (e.g. confidence/credible interval) and measures of statistical heterogeneity. If comparing groups, describe the direction of the effect. | 6-8 Fig 1-4 |  |
|  | 20c | Present results of all investigations of possible causes of heterogeneity among study results. | 6-7 |  |
|  | 20d | Present results of all sensitivity analyses conducted to assess the robustness of the synthesized results. | 6-7 |  |
| Reporting biases | 21 | Present assessments of risk of bias due to missing results (arising from reporting biases) for each synthesis assessed. | 6 |  |
| Certainty of evidence | 22 | Present assessments of certainty (or confidence) in the body of evidence for each outcome assessed. | 6-7 Fig 1-4 Supp Table S7 |  |
| **DISCUSSION** | | |  | |
| Discussion | 23a | Provide a general interpretation of the results in the context of other evidence. | 8-9 |  |
|  | 23b | Discuss any limitations of the evidence included in the review. | 9-10 |  |
|  | 23c | Discuss any limitations of the review processes used. | 10 |  |
|  | 23d | Discuss implications of the results for practice, policy, and future research. | 9-10 |  |
| **OTHER INFORMATION** | | |  | |
| Registration and protocol | 24a | Provide registration information for the review, including register name and registration number, or state that the review was not registered. | 1 |  |
|  | 24b | Indicate where the review protocol can be accessed, or state that a protocol was not prepared. | 1,2 |  |
|  | 24c | Describe and explain any amendments to information provided at registration or in the protocol. | 2 |  |
| Support | 25 | Describe sources of financial or non-financial support for the review, and the role of the funders or sponsors in the review. | 11 |  |
| Competing interests | 26 | Declare any competing interests of review authors. | 11 |  |
| Availability of data, code and other materials | 27 | Report which of the following are publicly available and where they can be found: template data collection forms; data extracted from included studies; data used for all analyses; analytic code; any other materials used in the review. | Supp Form S1 |  |

.

**Supplementary Table S2.** Literature search strategies and key words

| **Literature Database** | **Search strategy and keywords** |
| --- | --- |
| China National Knowledge Infrastructure | Search strategy  (TKA % '布拉氏酵母菌' +'布拉酵母菌' +'亿活' ) and (TKA % '腹泻' + '急性腹泻' + '轮状病毒性肠炎'+ '秋季腹泻'-'抗生素相关'-'幽门')  and (TKA % '小儿' + '儿童' + '婴幼儿'+ '新生儿'+ '患儿')  Keywords  布拉氏酵母菌、布拉酵母菌、亿活，腹泻、小儿急性腹泻、小儿腹泻、婴幼儿腹泻、新生儿腹泻、新生儿轮状病毒性肠炎、秋季腹泻、儿童腹泻、腹泻患儿  ["saccharomyces boulardii","Yihuo","diarrhea", "acute diarrhea", "rotavirus enteritis", "autumn diarrhea", "acute diarrhea in children", "infantile diarrhea","children diarrhea", "neonatal diarrhea", "diarrhea infants"] |
| China Biology Medicine disc | Search strategy #1"布拉氏酵母菌"[常用字段] OR "布拉酵母菌"[常用字段] OR "亿活"[常用字段] #2"腹泻"[常用字段] OR "急性腹泻"[常用字段] OR "轮状病毒性肠炎"[常用字段] OR "秋季腹泻"[常用字段] NOT "抗生素相关"[常用字段] NOT "幽门"[常用字段] #3"小儿"[常用字段] OR "儿童"[常用字段] OR "新生儿"[常用字段] OR "婴幼儿"[常用字段] OR "患儿"[常用字段] (#1) AND (#2) AND (#3) [translation: #1 “Saccharomyces boulardii" [common field] OR "Saccharomyces boulardii" [common field] OR "Billion Live" [common field] #2 "Diarrhea" [Common Field] OR "Acute Diarrhea" [Common Field] OR "Rotavirus Enteritis" [Common Field] OR "Autumn Diarrhea" [Common Field] NOT "Antibiotic Related" [Common Field] NOT "Pylorus" [Common Field] #3 "Child" [Frequently Used Field] OR "Child" [Frequently Used Field] OR "Newborn" [Frequently Used Field] OR "Infant" [Frequently Used Field] OR "Sick Child" [Frequently Used Field]  Keywords (same as above) but different strategy |
| Embase | 'pediatrics'/exp OR 'pediatrics') AND ('acute diarrhea'/exp OR 'acute diarrhea') AND ('china'/exp OR 'china') AND 'controlled trial' AND ‘Saccharomyces boulardii’ |
| Google Scholar | clinical trials AND acute pediatric diarrhea OR rotavirus AND Saccharomyces boulardii AND China NOT animals |
| PubMed | (“probiotics” [MeSH Terms] OR “probiotics” [All Fields]) AND [“pediatric” AND/OR “acute diarrhea” [MeSH Terms] AND “clinical trials” [All Fields] AND “China” [All Fields] AND “Saccharomyces boulardii” [All Fields]). |

**Supplementary Table S3**. Selected examples of excluded trials

| **Reference** | **Types of Probiotics compared** | **Reason excluded** |
| --- | --- | --- |
| Altcheh J 2022 | *S. boulardii* vs. *Bacillus clausii* | Trial done in Argentina |
| Duan W 2017 | *S. boulardii* vs. Bifido Quad. | Direct comparison of two probiotics, no non-probiotic control |
| Feng N 2018 | *S. boulardii* vs mezlocillin | Retrospective study |
| Li Gui-nan 2014 | *S. boulardii* vs standard treatment | Prevention of PAGE not treatment |
| Liu T 2020 | *S. boulardii* vs Bifido Quad | Direct comparison of two probiotics, no non-probiotic control |
| Mourey F 2020 | *S. boulardii* vs placebo | Strain *S. boulardii* CNCM I-3799 |
| Vineeth S 2017 | *S. boulardii* vs. *Bacillus clausii* | Trial done in India |
| Wang G 2019 | *S. boulardii* vs. *S. boulardii* + Bifido Triple | Control was *S. boulardii* with another probiotic type |
| Zhao YF 2017 | *S. boulardii* vs. *S. boulardii* + Bifido Triple | Control was *S. boulardii* with another probiotic type |

**References:**

Altcheh J, Carosella M V., Ceballos A, et al. Randomized, direct comparison study of *Saccharomyces boulardii* CNCM I-745 versus multi-strained Bacillus clausii probiotics for the treatment of pediatric acute gastroenteritis. Medicine (Baltimore) 2022;101(36):e30500. doi:10.1097/MD.0000000000030500

Duan W, Zhou C, Luo M, Zuo X. Effects of *Saccharomyces boulardii* powder on disease progression of rotavirus gastroenteritis in children. Mod Dig Inter. 2017;22(5):692-694. doi:10.3969/j.issn.1672-2159.2017.05.028

Feng N, Lei Z, Yang H, Hu L. Clinical efficacy of *Saccharomyces boulardii* combined with mezlocillin in the treatment of children with infectious diarrhea and effect on serum CRP,PCT and IL-8. Chinese J Integr Tradit West Med Dig 2018;26(2):194-197.

Li G, Wu Y, Li J, et al. Clinical research of using *Saccharomyces boulardii* to prevent secondary diarrhea in hospitalized neonates. Chin J Microecol 2014;26(1):1-3.

Liu T. Analysis of the clinical effect of treating children with rotavirus gastroenteritis with *Saccharomyces boulardii* and Bifidobacterium quadruple live bacteria. Cardiovasc Dis Integr Tradit Chinese West Med 2020;8(34):57-63. doi:10.16282/j.cnki.cn11-9336/r.2020.34.040

Mourey F, Sureja V, Kheni D, et al. A Multicenter, Randomized, double-blind, placebo-controlled Trial of *Saccharomyces boulardii* in infants and children With acute diarrhea. Pediatr Infect Dis J 2020;39(11):e347-e351. doi:10.1097/INF.0000000000002849

Vineeth S, Saireddy S, Keerthi T, Mantada PK. Efficacy of *Bacillus clausii* and *Saccharomyces boulardii* in treatment of acute rotaviral diarrhea in pediatric patients. Indonesian Journal of Clinical Pharmacy 2017; 6(2):91-98. doi: 10.15416/ijcp.2017.6.2.91

Wang G, Feng D. Therapeutic effect of *Saccharomyces boulardii* combined with *Bifidobacterium* and on cellular immune function in children with acute diarrhea. Experimental and Therapeutic Medicine 2019;18(4):2653-9.

Zhao YF, Shao X, Xu B, et al. Influence of *Saccharomyces boulardii* on expression of serum IL-6 and TNF-α of children with rotavirus infections. Chinese J Nosocomiology 2017;27(21):4989-4991. doi:10.11816/cn.ni.2017-170-946.

**Table S4.** Study population and intervention characteristics for trials with pediatric acute gastroenteritis (PAGE) comparing *Saccharomyces boulardii* CNCM I-745 and controls.

| **Reference** | **N enrolled** | **Age range** | **Days diarrhea prior to enrollment** | ***S. boulardii* dose (mg/d)** | **Duration study treatment (days)** | **Type of patient (inpatient or outpatient)** | **Adverse event** |
| --- | --- | --- | --- | --- | --- | --- | --- |
| Cao SX 2017 [32] | 188 | 3 mon- 5 y | < 3 d | Nr | 4 | Inpatient | None |
| Chen LL 2014 [33] | 84 | <1.4 y | < 3 d | <1 y: 125 mg >1 y: 500 mg | 3 | Outpatient | None |
| Chen QJ 2020 [34] | 98 | 2 mon-2 y | < 3 d | <1 y: 125 mg >1 y: 500 mg | 7 | Inpatient | 4% vs 18% |
| Lv CG 2014 [35] | 85 | 6 mon- 6 y | < 2 d | <1 y: 250 mg >1 y: 500 mg | 3 | Inpatient | None |
| Qiu HM 2018 [36] | 96 | 3 mon- 3 y | < 15 d | 500 mg | 3 | Inpatient | Nr |
| Qu YH 2012 [37] | 110 | 3 mon- 5 y | acute | <1 y: 250 mg >1 y: 500 mg | 7 | Inpatient | None |
| Tan HM 2015 [38] | 110 | 0 mon- 3 y | < 2 d | <1 y: 125 mg >1 y: 250 mg | 3 | Inpatient | None |
| Wu ZL 2021 [39] | 102 | 7 mon- 5 y | acute | 500 mg | Nr | Inpatient | 2% vs 16% |
| Yang XH 2015 [40] | 96 | 4 mon- 1.5 y | 1-5 | <1 y: 125 mg >1 y: 500 mg | 7 | Inpatient | Nr |
| Yao LY 2018 [41] | 156 | <2 y | < 3 d | <1 mon: 250 mg >1 mon: 500 mg | 5 | Inpatient | 3%-13% |

**Abbreviations:** Acute, specific day on onset not reported; High, high risk of bias; mg, milligram; mon, month; Nr, not reported; mon, months; SC, some concerns for bias; vs, versus; y, years.

**Supplementary Table S5.** Risk of bias by domains for each included trial in children with PAGE.

| **Ref** | **Randomization method** | **Bias control** | **Missing outcome data** | **Outcome measures** | **A prior outcomes** | **Overall score** |
| --- | --- | --- | --- | --- | --- | --- |
| Cao SX 2017 [32] | Low | High | Low | Low | Low | Some concerns |
| Chen LL 2014 [33] | Low | High | Low | Low | Low | Some concerns |
| Chen QJ 2020 [34] | Low | High | Low | Low | Low | Some concerns |
| Lv CG 2014 [35] | Low | High | Low | Low | Low | Some concerns |
| Qiu HM 2018 [36] | Low | High | Low | Low | Low | Some concerns |
| Qu YH 2012 [37] | Low | High | Low | Low | Low | Some concerns |
| Tan HM 2015 [38] | High | High | Low | Low | Low | High |
| Wu ZL 2021 [39] | Low | High | Low | Low | Low | Some concerns |
| Yang XH 2015 [40] | Low | High | Low | Low | Low | Some concerns |
| Yao LY 2018 [41] | Low | High | Low | Low | Low | Some concerns |

**Supplementary Table S6**. Changes in inflammatory marker levels comparing *S. boulardii* CNCM I-745 with controls in children with PAGE.

| **Ref** | **Changes in TNF-α levels, Sb (pg/ml)** | **Changes in TNF=-α levels, controls (pg/ml)** | **Change in  IL-8 Sb** | **Change in IL-8 controls  (mg/L)** | **Change in CD4/CD8 ratio, Sb** | **Change in CD4/CD8 ratio, controls** | **Other type of inflammatory markers** |
| --- | --- | --- | --- | --- | --- | --- | --- |
| Cao SX 2017 [32] | Nr | Nr | Nr | Nr | +0.55 + 0.23* | +0.06 + 0.09 | CD3* |
| Chen LL 2014 [33] | -1.79 + 0.31* | - 1.07 + 0.14 | Nr | Nr | Nr | Nr | IL6** |
| Chen QJ 2020 [34] | -2.06 + 0.22* | -0.76 + 0.66 | -94.8 + 16.8* | -60.98 + 15.0 | Nr | Nr | IL6** |
| Lv CG 2014 [35] | Nr | Nr | Nr | Nr | Nr | Nr | Nr |
| Qiu HM 2018 [36] | Nr | Nr | - 17.3 +0.05* | -13.3 + 0.06 | Nr | Nr | CRP**, IL10* |
| Qu YH 2012 [37] | Nr | Nr | Nr | Nr | Nr | Nr | Nr |
| Tan HM 2015 [38] | Nr | Nr | Nr | Nr | Nr | Nr | Nr |
| Wu ZL 2021 [39] | Nr | Nr | Nr | Nr | Nr | Nr | Nr |
| Yang XH 2015 [40] | -1.98 + 0.38* | --1.11 + 0.25 | -95.8 + 17.2* | -61.5 + 13.3 | Nr | Nr | Nr |
| Yao LY 2018 [41] | Nr | Nr | Nr | Nr | Nr | Nr | Nr |

*Significant increase, ** Significant decrease, P<0.05

**Supplementary Table S7.** GRADE recommendations

| **Outcome** | **Estimated effect in *S. boulardii* compared to controls** | **Number of participants (studies)** | **Certainty of evidence  (GRADE)** |
| --- | --- | --- | --- |
| Duration of diarrhea | SMD: -1.63 days, CI: -2.08, -1.18, I^2^=85% | 750 (6) | Low ^a,d,e^ |
| Total Effectiveness Rating | RR= 1.22, CI: 1.16, 1.28, I^2^=0% | 1125 (10) | Moderate ^a^ |
| Cured | RR=1.47, CI: 1.30, 1.67, I^2^=30.9% | 1125 (10) | Moderate ^a^ |
| Reduction TNF-α | SMD: -2.76, CI: -3.09, -2.43, I^2^=0% | 278 (3) | Low ^a,e^ |
| Reduction in IL-8 | SMD: -11.2, CI: -15.6, -6.8, I^2^=98.9% | 290 (3) | Low ^a,e^ |

**Abbreviations**: CI, 95% confidence interval; I^2^, measure of heterogeneity; RR, relative risk; SMD: standardized mean difference

**GRADE definitions of evidence:**High certainty: Very confident that the true effect lies close to that of the estimate of the effect

Moderate certainty: Moderately confident in the effect estimate. The true effect is likely to be close to the estimate of the effect, but there is a possibility that it is substantially different.

Low certainty: Confidence in the effect is limited. The true effect may be substantially different from the estimate of the effect.

Very low certainty: Very little confidence in the effect estimate. The true effect is likely to be substantially different from the estimate of effect.

**Notes:**^a^ All trials were unblinded

^b^ Substantial heterogeneity (I^2^>90%) partially explained by subgroup analysis

^c^ Substantial heterogeneity (I^2^>90%) not explained by subgroup analysis

^d^ High heterogeneity (I^2^>50%) partially explained by subgroup analysis (etiology)

^e^ Based on low numbers of trials

**Supplementary Form S1.** Data Extraction Form.

| **Item** | **Score Rev #1** | **Score Rev #2** | **Item** | | | **Source** |
| --- | --- | --- | --- | --- | --- | --- |
| 1 |  |  | Randomized controlled trial | | |  |
| 2 |  |  | Background | | |  |
| 3 |  |  | Aim(s) | | |  |
| 4 |  |  | Setting: China | | |  |
| 5 |  |  | Eligibility/exclusions | | |  |
| 6 |  |  | Sample size calculation | | |  |
| 7 |  |  | Statistical methods described | | |  |
| 8 |  |  | Participants (peds, in/out, age range, total N=  Days diarrhea before enrollment: Sb: or control: | | |  |
| 9 |  |  | Randomization method | | |  |
| 10 |  |  | Allocation method | | |  |
| 11 |  |  | Blinded (0=open, 1-single/double) | | |  |
| 12 |  |  | Outcome assess blind | | |  |
| 13 |  |  | Attrition rates given: no attrition | | |  |
| 14 |  |  | Only a priori outcomes (no new ones) | | |  |
| 15 |  |  | Intervention: Sb strain (brandname/Biocodex/import #)  Daily dose  Formulation: __ powder  Duration  Follow-up duration (post-Sb) | | |  |
| Not scored- information only | | | Types of Standard Therapies to all:  Diet, oral or IV rehydration, antivirals or antibiotics as needed | | |  |
| 16 |  |  | Baseline data compared | | |  |
| 17 |  |  | Consort flowchart (>2006) | | |  |
| 18 |  |  | 1^o^ outcome defined/how documented: | | |  |
| 19 |  |  | 1^o^ outcome data:   duration diarr  cured by end  total effective rate  #bm/day on Day __ 2^o^ outcomes  immune markers Subgroups:  duration SB  dose  risk bias  etiology | SB | control |  |
| 20 |  |  | AE data given or just statement “No AE seen” | | |  |
| 21 |  |  | Limitations | | |  |
| 22 |  |  | Generalizability | | |  |
| 23 |  |  | Other studies | | |  |
| 24 |  |  | registered | | |  |
| R#1 |  |  | Total items scored: +1: 0: na: Total: | | |  |
| R#2 |  |  | Total items scored: +1: 0: na: Total: | | |  |

| **Item** | **Domain** | **Items** | **Reviewer #1** | **Reviewer #2** |
| --- | --- | --- | --- | --- |
| 1 | Randomization process | Randomized/ baseline same/ allocation blinded |  |  |
| 2 | Bias control | staff blinded/patient blinded/revisions did not effect outcome/same for each group |  |  |
| 3 | Missing outcome data | most outcome data reported/low attrition or same/group |  |  |
| 4 | Outcome measurement | Appropriate measures/Measures same by group/Outcome assessor blinded |  |  |
| 5 | Reported outcomes same as initial protocol | Reported outcomes defined a priori/No new post-hoc outcomes |  |  |
| 6 | Overall | Low=all (1-5) scored low  Some Concerns: 1 scored high risk  High: >2 scored high |  |  |
| Scoring: Each domain: Low=more factors present than absent, High=more absent | | | | |
